# Analysis of Postoperative Complication and Revision Rates and Mid- to Long-Term Implant Survival in Primary Short-Stem Total Hip Arthroplasty

**DOI:** 10.3390/jcm13133779

**Published:** 2024-06-27

**Authors:** Ricarda Stauss, Nils T. Becker, Peter Savov, Max Ettinger, Gesine H. Seeber

**Affiliations:** 1 Division of Orthopaedics at Campus Pius-Hospital, School of Medicine and Health Sciences, Carl von Ossietzky Universität Oldenburg, 26121 Oldenburg, Germany; 2 Department of Orthopedics, University Medical Center Groningen, University of Groningen, P.O. Box 30.001, 9700 RB Groningen, The Netherlands

**Keywords:** total hip arthroplasty, short stem, complication rate, revision rate, implant survival

## Abstract

**Background/Objectives:** Short-stem prostheses were introduced as an alternative to conventional straight-stem prostheses. Despite their benefits, including minimally invasive approaches, soft-tissue- and bone-sparing implantation, and physiological load transfer to the metaphysis, data on postoperative complication and revision rates as well as on implant survival are scarce. **Methods:** A retrospective analysis of 1327 patients who underwent primary total hip arthroplasty (THA) using the Metha^®^ short stem between 2006 and 2023 was conducted. Complication and revision rates were analysed for the intraoperative, direct postoperative, and follow-up episodes. Implant survival was analysed with the endpoint of all-cause stem revision. **Results:** Intraoperative complications were observed in 3.77% of the cases and included 44 hairline cracks and 6 fractures. In 15 cases (30.0%), conversion to a straight-stem or revision implant was necessary. The direct postoperative complication rate was 2.44%, and 11 revision procedures were performed during inpatient stay (0.84%). Mean follow-up was 7 years (range 1–17). During follow-up, femoral component revision was performed in 60 cases. Aseptic loosening and stem subsidence accounted for a combined percentage of 80% of all indications. Implant survival rate was 95.66% after 5 years, 95.58% after 10 years, and 95.50% after 15 years. **Conclusions:** Our study provides a comprehensive analysis of postoperative complication and revision rates in a large sample undergoing primary short-stem THA. Postoperative complication rates were favourable, and the long-term implant survival rates were comparable to conventional straight-stem prostheses. Therefore, short-stem THA may be considered an alternative for younger patients.

## 1. Introduction

Total hip arthroplasty (THA) is the standard treatment for end-stage osteoarthritis (OA) of the hip. It is considered the “operation of the century”, as it is one of the most successful orthopaedic procedures, leading to substantial improvements in patients’ quality of life [1]. Given the high success rates of modern THA procedures, the volume of primary THA has risen exponentially in the past years [2,3]. Recent models predict a 5% annual growth in THA procedures and forecast an increase in primary THA procedures of 600% by 2060 [3]. In addition, arthroplasty registries indicate an increasing proportion of young, high-demanding, and physically active patients undergoing THA [4,5,6]. Kurtz et al. predicted that by the year 2030, 52% of all primary THAs would be performed in patients younger than 65 years of age, with the greatest increase in patients aged 45–55 years [4]. Given the average implant survival rate of 89% after 15 years and 58% after 25 years, these patients are at a higher risk for consecutive revision procedures [7]. In accordance with this, Bayliss et al. report a significant 29% increase in the lifetime risk of revision (LTRR) for patients aged 50–54 years versus a LTRR of 5% in patients aged 70 years [8].

Despite the favourable long-term outcomes of conventional cementless straight-stem prostheses, there are relevant limitations of these stem designs, including an unphysiological distal load transfer, stress shielding with consecutive periprosthetic bone loss in the proximal femur, and thigh pain due to the diaphyseal anchorage [9,10,11].

Short-stem implants were introduced as a soft-tissue- and bone-preserving alternative to conventional straight-stem THA and have been gaining popularity ever since. They allow for minimally invasive surgical approaches and physiological loading by distributing stress forces solely to the femoral metaphysis, thereby reducing periprosthetic bone remodelling [12,13]. However, data on the postoperative complication rates, long-term outcomes, and survival rates of short stems are scarce.

The purpose of this study was to examine the intra- and postoperative complications and revision rates associated with short-stem THA and to analyse mid- and long-term implant survival rates of the Metha short-stem prosthesis.

## 2. Materials and Methods

This is a retrospective, single-centre study investigating the intra- and postoperative complication and revision rates using the Metha^®^ short-stem prosthesis (BBraun, Aesculap, Tuttlingen, Germany). Following ethical approval, the institutional database at our tertiary referral centre was screened to identify patients who had undergone THA using the Metha short-stem prosthesis between November 2006 and November 2023. In general, indications for short-stem arthroplasty included younger patient age (<70 years), good femoral bone stock, absence of severe deformities, and a proximal femoral morphology that allows for primary stability of a metaphyseal-anchoring implant. Exclusion criteria were defined as age < 18 years, incomplete digital medical records, and use of the Metha short stem in revision THA (rTHA). In accordance with the aforementioned inclusion and exclusion criteria, 1327 cases were deemed eligible for analysis.

### 2.1. Implant Characteristics

The Metha short stem is a calcar-loading, partial neck-preserving implant [14]. Metaphyseal anchorage is achieved by the proximal, trapezoidal implant design, which allows for a cortical multipoint contact and a three-point fixation in the medial calcar region, proximal lateral cortex, and proximal posterior cortex [15,16].

The Metha short-stem prosthesis was initially introduced in 2004 as a modular stem with titanium alloy neck adapters (CCD angle specifications: 130°, 135°, 140°; version options: neutral, 7.5° anteversion, 7.5° retroversion). In 2007, the adapter was replaced by a cobalt–chromium alloy modular neck adapter after a series of adapter failures of the titanium alloy adapter had been reported [17,18,19]. In 2008, the monoblock version was introduced (CCD angle specifications: 120°, 130°, 135°). Both stem designs are available in eight sizes (sizes 0–7).

### 2.2. Surgical Procedure

Preoperative templating was conducted to assess proximal femoral and acetabular anatomy, plan the osteotomy location, and predict component size and implant position [20]. All patients received prophylactic single-shot antibiotics perioperatively. Surgeries were performed using an anterolateral, lateral, or direct anterior approach with the patient in a supine position. For cup placement, an image-free navigation system was used (OrthoPilot^®^, BBraun Aesculap, Tuttlingen, Germany). Following an intraoperative assessment of the bone quality of the proximal femur, stem preparation and implantation were conducted according to the manufacturer’s instructions. For femoral neck osteotomy, a neck-preserving resection is mandatory to ensure a sufficient proximal anchoring of the prosthesis and specific care is taken to ensure an intact cortical ring of the femoral neck, which is crucial for primary stability. During femoral preparation, specific care is taken to achieve rotational stability within the cortical ring and a sufficient contact with the dorsolateral cortex. Radiographs were taken intraoperatively to confirm the correct implant position.

### 2.3. Clinical Data

Demographic, clinical, and intraoperative data were retrieved from the digital medical records. Postoperative complications and revision surgeries were documented during clinical follow-up and were analysed at last follow-up. Arthroplasty-related postoperative complications were defined according to Healy et al. and classified as intraoperative, direct postoperative complications during inpatient stay, and late postoperative complications that occurred during the follow-up period after discharge [21]. The primary endpoint was defined as femoral component revision for any reason.

### 2.4. Statistical Analysis

Descriptive statistics were calculated including counts and frequencies for categorical data. For continuous data, means and standard deviations (SDs) or medians and interquartile ranges (IQRs) are provided. Group differences were calculated using Student’s *t*-test or the Mann–Whitney U-test. The Kruskal–Wallis test was used for multiple-group comparisons. Bonferroni adjustment was applied for multiple testing. A non-parametric survival analysis was conducted using Kaplan–Meier calculations (observed cumulative survival). Implant survival was defined as the time between primary THA to first revision (event). Patients who did not undergo rTHA were censored at the end of the study period (i.e., 30 November 2023) or at the time of in-hospital death. Subgroups were compared using the log rank test to assess the impact of stem design on implant survival. A *p*-value < 0.05 was considered statistically significant. Statistical analysis was performed using IBM SPSS Statistics 29 (SPSS Inc., Armonk, NY, USA).

## 3. Results

Of 1327 patients being included into this study, 725 were female (54.63%). Mean age at the time of surgery was 55 years. Primary OA accounted for 56% of the cases. Baseline characteristics are provided in Table 1.

**Figure 1:**
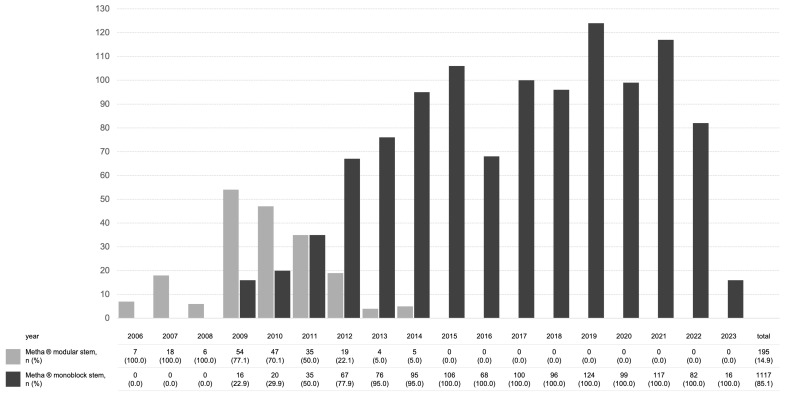
Overview of implanted stem designs over the years.

The monoblock stem was used in 84% of cases (Table 2). The modular stem was available from 2006 and was used until 2014; however, the proportion of monoblock stems increased steadily since their introduction in 2008 (Figure 1). An optical navigation system was used for cup placement in 98% of cases.

### 3.1. Inpatient Stay

**Figure 2:**
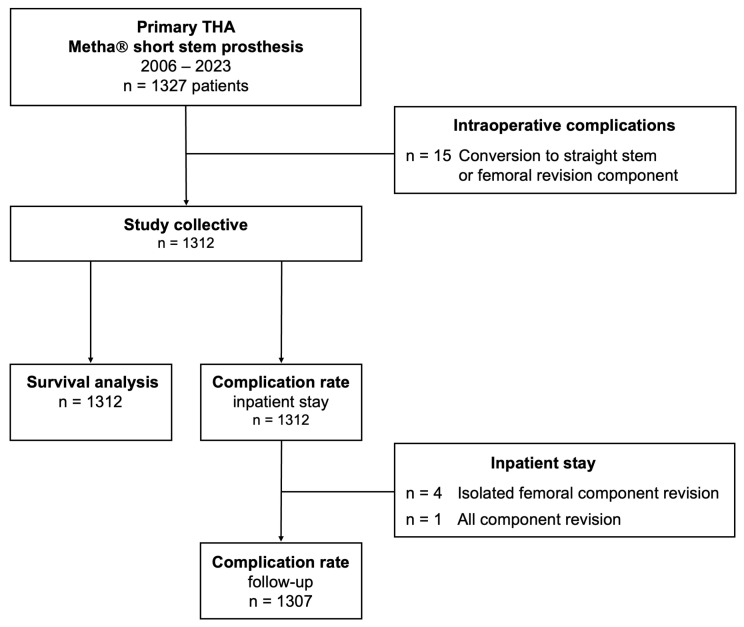
Flowchart of the study design. THA—total hip arthroplasty.

Intraoperative complications were detected in 50 cases (3.77%) and included 44 hairline cracks (3.32%) and 6 intraoperative periprosthetic femur fractures (0.45%) (Table 3). While conservative management was possible in 22 out of 50 cases (44.0%), cerclage cabling was performed in 13 cases (26.0%). In 15 cases (30.0%), intraoperative conversion to a straight stem with diaphyseal anchorage or a femoral revision implant was necessary. These cases were excluded from subsequent analyses (Figure 2).

Direct postoperative complications during inpatient stay were detected in 32 cases, resulting in a complication rate of 2.44% (Table 4). In total, 11 revision procedures were performed during inpatient stay, representing a revision rate of 0.84%. Mean time to revision was 8 days (range 0–12 days). Revision procedures included superficial wound revision (n = 3) and postoperative haematoma evacuation (n = 2). Reasons for early THA revisions were inlay dislocation leading to an isolated head and liner exchange (n = 1; 0.08%); isolated femoral component revisions due to early stem subsidence (n = 2, 0.15%); leg length discrepancy (n = 1, 0.08%); femoral fracture (n = 1, 0.08%); and acetabular component dislocation leading to all component revision (n = 1; 0.08%).

Stratified by patient age, overall intraoperative and early postoperative complication rates did not differ significantly between the groups (*p* = 0.607, *p* = 0.978).

No statistically significant group differences were observed for intraoperative and early postoperative complication rates stratified by surgical approach (*p* = 0.097, *p* = 0.253) or stem design (*p* = 0.429, *p* = 0.482).

### 3.2. Follow-Up

Detailed information on surgery-related complications during the follow-up period is provided in Table 5. Median follow-up period was 86 months (equivalent to approximately 7 years). Of all patients, 857 (65.6%) had a follow-up of minimum 5 years, 386 (45.0%) had a follow-up of minimum 10 years, and 26 (2.0%) had a follow-up of minimum 15 years.

Postoperative THA-related complications were observed in 89 cases, resulting in a respective complication rate of 6.81%. Overall, complication rates during follow-up did not reveal statistically significant differences between the groups stratified by age (*p* = 0.636).

Periprosthetic infection was detected in three cases (0.23%), including one acute and one chronic PJI. Mechanical complications following primary short-stem THA accounted for a combined percentage of 5.43%, including three cases of femoral neck adapter fracture (n = 2, 0.15%) or corrosion (n = 1, 0.08%). Stem subsidence was recorded in 44 cases, leading to isolated femoral component revision in 20 cases (1.53%). Periprosthetic femur fractures occurred in five cases (0.38%), of which two Vancouver B1 fractures were treated with open reduction and internal fixation (ORIF), while one case required femoral component revision using a diaphyseal anchoring revision implant.

During the follow-up period, 60 visits to our outpatient clinic were registered due to a prolonged postoperative course with residual functional limitations and hip pain without a mechanical correlate. However, none of these cases resulted in THA revision.

### 3.3. Survival Analysis

In total, 77 THA revision procedures were performed during inpatient stay and the follow-up period. In 60 cases, the femoral component was revised (Table 6). The majority of femoral stem revisions were attributable to aseptic loosening of the femoral component (46.67%) and stem subsidence (30.00%). Neck adapter failure of the modular stem was observed in 3 out of 195 cases (1.54%). In one case, the reason for rTHA remained unclear, as the surgical procedure was performed in another hospital. Table 6 provides detailed information on the indications leading to femoral component revision.

In 25 cases (41.67%), rTHA was performed using a short stem as a revision component. In all of these cases, a larger stem was used and the mean increase in stem size was three sizes (range 1–4 sizes). Femoral stem revision to another primary implant, including anatomic or straight-stem prostheses according to the classification system proposed by Kheir et al. [22], was conducted in 32 cases (53.33%). In one case (1.67%), a femoral revision implant was used.

**Figure 3:**
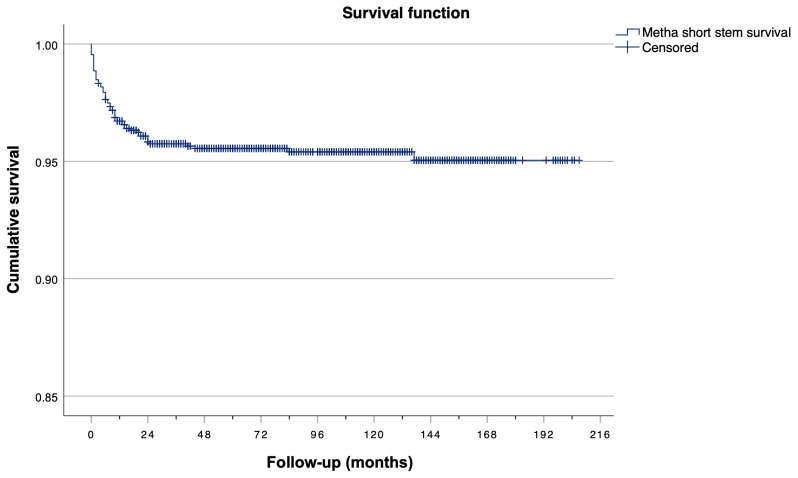
Kaplan–Meier curve for overall stem survival of the modular stem and monoblock stem combined.

**Figure 4:**
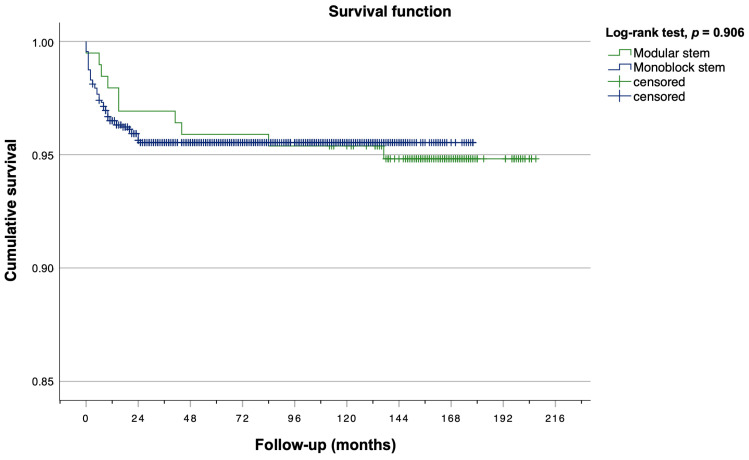
Kaplan–Meier survival plot for femoral stem revision stratified by stem subgroup.

Kaplan–Meier analysis revealed a 5-year survival rate of 95.66% (57/1312), a 10-year survival rate of 95.58% (58/1312), and a 15-year survival rate of 95.50% (59/1312) for both the modular stem and the monoblock stem combined (Figure 3). Moreover, Kaplan–Meier estimators were calculated separately for monoblock and modular stems (Figure 4). No statistically significant between-group differences were observed regarding stem survival (log rank test, *p* = 0.906). The modular stem exhibited a 5-year survival rate of 95.90% (8/195) and a 10-year survival rate of 95.38% (9/195). For the monoblock stem, 5-year and 10-year survival rates were 96.15% (43/1117) and 95.61% (49/1117), respectively. The most recent stem revision was conducted 25 months after the index surgery.

## 4. Discussion

The most important finding of this study is that the Metha short-stem prosthesis can achieve comparable long-term survival rates to those of conventional straight-stem prostheses. Moreover, this study demonstrated a low incidence of intra- and postoperative complications and a low rate of required revision surgeries. These findings lend support to the hypothesis that the Metha short-stem prosthesis may be a viable alternative to conventional straight-stem prostheses especially in young patients undergoing THA surgery.

The incidence of intraoperative periprosthetic fractures (IPFF) in primary cementless THA differs, ranging from 0.4% to 6.8% [23,24,25,26]. However, for cementless THA, IPFF rates of up to 28% have been reported in older studies in the literature [27,28]. In our study population, an IPFF rate of 0.45% was observed. This is in line with evidence from previous studies reporting IPFF rates for short-stem THA of 0.8% and 0.4%, respectively [23,25]. Furthermore, we report a total count of 44 intraoperative periprosthetic fissures and calcar cracks, representing 88% of all intraoperative complications. This is consistent with data from the National Joint Registry for England and Wales published in 2019 [29], where calcar cracks were the most common IPFF subtype and the risk of intraoperative calcar cracks was highest in the youngest age group (50–59 years). In contrast, the risk of shaft fractures significantly increased in patients aged more than 80 years. Previous studies revealed an association between femoral canal shape and demographic characteristics [30,31,32]. Specifically, lower age is associated with higher cortical thickness and Dorr type A canal shape, whereas Dorr type C femora, characterised by thin cortices and a wide intramedullary canal, are associated with higher age. Consequently, prolonged and more forceful rasping during femoral canal preparation may be required in younger patients, thus contributing to higher IPFF rates in these patients.

We observed a cumulative postoperative periprosthetic fracture (PPFF) rate of 0.46% during inpatient stay and follow-up, which is lower than previously published evidence on PPFF in short-stem THA. Luger et al. found a significant group difference of PPFF rates in short-stem versus straight-stem arthroplasty (1.7% vs. 3.2%) [25]. Nelson et al. report lower 90-day PPFF rates for short-stem (0.31–1.04%) compared to straight-stem implants (1.22%) and the authors were able to prove non-inferiority for 90-day fracture rate in the short-stem subgroup [33]. However, it must be mentioned that the comparability of these results is limited by the varying follow-up periods reported in these studies. Moreover, it has to be kept in mind that differences in fracture rates may be biased by the different demographic characteristics of the short-stem and straight-stem patient populations. While patients undergoing short-stem THA are often younger and more physically active, older age, the female sex, and poor bone quality are associated with higher risks of PPFF [24,34].

The rate of THA-related complications during inpatient stay was 2.44%. leading to early THA revision in 0.46% of cases. This is in line with evidence from Huang et al., who report a THA-related complication rate of 2.12% during inpatient stay and a revision rate of 1.15% [35]. In the literature, early complications following THA include venous thromboembolism, PJI, and mechanical complications including instability or dislocation, LLD and fractures [36,37]. In our study, early complications also included postoperative wound complications or haematoma leading to surgical revision and temporary neural deficits caused by intraoperative retractor placement. However, as there is no consensus on the definition of early THA revisions and the analysed episodes differ remarkable between the studies, the generalisability of different studies’ results is constrained [38,39,40,41]. For future research, it is essential to establish standards for the reporting of early THA-related complications.

During follow-up, THA-related complications were observed in 6.81% of the cases. In our cohort, mechanical complications accounted for a combined percentage of 5.43%. Previously published studies based on large national databases in the US identified instability or dislocation as the most common reason for rTHA (17.3–19.3%), followed by mechanical loosening (16.8–19.7%) and infection (12.8–17.3%) [42,43,44]. Notably, in our study population, instability and dislocation were observed in only 0.30% of patients, which may be attributable to the fact that a navigation system was used for cup placement in 98% of the cases. Moreover, prior studies investigated the reconstruction of hip biomechanics and functional outcomes using a short stem versus conventional stem and revealed promising results for the reconstruction of individual hip anatomy using cementless short-stem arthroplasty [45,46,47,48]. In our study, aseptic stem or acetabular cup loosening were the most common THA-related complications, with aseptic loosening of the femoral component being the predominant reason for stem revision (46.67%), followed by stem subsidence (30.00%). This is in line with evidence from recent studies. In a study by Schwarz et al., aseptic loosening accounted for 40.87% of rTHA procedures [40]. Furthermore, Markel et al. found significant differences in causes for rTHA over time and a steady increase in the incidence of aseptic loosening, as it accounted for 20.5% of rTHA performed within 6–12 months postoperatively and for 31.9% of rTHA performed beyond one year post index procedure. [38] Given the proportion of femoral stem revisions attributable to aseptic loosening and the relatively high lifetime risk of revision for young patients undergoing THA, short stems may be considered a bone-preserving alternative to straight-stem implants in a younger patient population.

Implant survival rates were 95.7%, 95.6%, and 95.5% after 5, 10, and 15 years, respectively, with the primary endpoint of femoral stem revision. The revision rate observed in our study population was higher than those previously reported. Weenders et al. observed a 2.5% ten-year revision rate for the monoblock stem [49], and Schnurr et al. reported a 4.1% all-cause revision rate after a mean follow-up of 6 years and a 7-year stem revision rate of 1.5 and 1.8% for the monoblock and modular stem, respectively [15]. A recent review by Malahias et al. showed a 2.5% revision rate and found failure of the modular neck adapter to be the most common reason for revision [50]. Of note, only three neck adapter failures (0.23%) occurred in our population, which is lower than previously reported rates of up to 6% [15,17,51]. Interestingly, in our study sample, nearly all femoral component revisions were performed within the first two years following the index procedure. As aseptic loosening and stem subsidence were the leading causes for stem revisions, a mismatch between proximal femoral anatomy and implant geometry may be a possible explanation. This hypothesis is supported by evidence from previous studies, which showed an association between early subsidence and increased revision rates due to aseptic loosening [52], and identified undersizing as one of the main reasons for early stem failure [53,54]. In our study cohort, the mean increase of stem size in rTHA using another short-stem implant was three sizes, which may support this finding. However, as no radiological follow-up was conducted, this remains hypothetical. A comprehensive radiological analysis of femoral morphology and femoral component filling, as well as a thorough investigation of the radiological signs of implant failure, would be of value.

The current study exhibits several noteworthy strengths, including large sample size, long-term follow-up, and comprehensive data on THA-related intra- and postoperative complications and revision rates. However, it is essential to acknowledge the study is not without limitations. First, it is limited by its retrospective and single-centre design, as both aspects introduce a risk of bias. Due to the retrospective design, no clinical follow-up was conducted, and consequently, some cases may have been lost to follow-up. However, this limitation may be offset by the fact that most THA-related complications are readmitted to the primary institution, especially given that this study was conducted in a tertiary referral centre. Second, the results presented in this study are based on complication and revision rates only, and patient-reported outcome measures were not available. Third, no radiological data are presented. Future studies are warranted to examine the radiographic changes following short-stem THA in a longitudinal study design including radiological signs of bone remodelling, aseptic loosening, or stem subsidence.

## 5. Conclusions

In conclusion, our study provides a comprehensive analysis of postoperative complication and revision rates in a large sample undergoing cementless primary THA using the Metha short-stem prosthesis. Postoperative THA-related complication rates were favourable, and the implant survival rate of 96% at ten years is promising. However, as a relevant proportion of stem failures was observed within two years after the index procedure, future studies are required to investigate early radiological outcomes and implant positioning including the analysis of proximal femoral anatomy and the canal fill ratio or modified neck fill ratio of short-stem implants as well as clinical and radiological long-term outcomes.

## Figures and Tables

**Table 1:** Baseline characteristics.

				Study Sample (n = 1327)	
Age (y), mean (SD)				54.6	(±9.13)
Age categories, n (%)					
	Age < 60 years			883	(66.54)
	Age ≥ 60 years			304	(22.91)
	Age ≥ 65 years			128	(9.65)
	Age ≥ 70 years			11	(0.83)
	Age ≥ 75 years			1	(0.08)
Sex, n (%)					
	Male			602	(45.37)
	Female			725	(54.63)
BMI (kg/m^2^), mean (SD)				28.8	(±5.21)
ASA-Score, n (%)					
		1		117	(8.82)
		2		889	(66.99)
		3		173	(13.04)
		Missing data		148	(11.15)
Aetiology, n (%)					
	Primary OA			744	(56.07)
	Secondary OA			583	(43.93)
			Developmental dysplasia of the hip	384	(28.94)
			Avascular necrosis	130	(9.80)
			Posttraumatic OA	20	(1.51)
			History of LCPD	18	(1.36)
			History of SCFE	17	(1.28)
			Rheumatoid arthritis	8	(0.60)
			FAI	4	(0.30)
			History of coxitis	2	(0.15)

ASA—American Society of Anesthesiologists; BMI—Body Mass Index; FAI—femoroacetabular impingement; OA—osteoarthritis; LCPD—Legg–Calvé–Perthes disease; SCFE—slipped capital femoral epiphysis; SD—standard deviation.

**Table 2:** Intraoperative data.

		Study Sample (n = 1327)	
Implant design, n (%)			
	Metha modular stem	195	(14.69)
	Metha monoblock stem	1117	(84.17)
	Other ^1^	15	(1.13)
Surgical technique, n (%)			
	Optical navigation system	1305	(98.34)
	Conventional manual technique	22	(1.66)
Surgical approach, n (%)			
	Anterolateral	978	(73.70)
	Lateral	246	(18.54)
	Direct anterior	53	(3.99)
	Missing data	50	(3.77)

^1^ Intraoperative conversion to a straight-stem prosthesis with diaphyseal anchorage or femoral revision implant in case of an intraoperative periprosthetic fracture.

**Table 3:** Intraoperative complications.

			Study Sample (n = 1327)	
Intraoperative complications, n (%)			50	(3.77)
	Hairline cracks		44	(3.32)
	Periprosthetic fracture		6	(0.45)
		Vancouver AG	1	(0.08)
		Vancouver B1	1	(0.08)
		Vancouver B2	4	(0.30)
Surgical management, n (%)				
	Conservative management		22	(44.00)
	Cerclage wires		13	(26.00)
	Stem replacement with straight-stem or femoral revision implant		15	(30.00)

**Table 4:** Surgery-related complications and revision procedures during inpatient stay.

			Study Sample (n = 1312)	
Surgery-related complications during inpatient stay, n (%)			32	(2.44)
Wound complication requiring revision			3	(0.23)
Postoperative haematoma requiring surgical treatment			3	(0.23)
Mechanical complications			11	(0.84)
	Stem subsidence requiring revision		2	(0.15)
	Periprosthetic fracture		1	(0.08)
	Acetabular cup loosening		1	(0.08)
	Cup-liner dissociation		1	(0.08)
	Instability/dislocation		2	(0.15)
	Leg length discrepancy		1	(0.08)
Temporary neural deficit			14	(1.07)
Deep vein thrombosis			1	(0.08)
Revision procedures during inpatient stay, n (%)			11	(0.84)
Mean time to revision, days (SD)			7.60	(±4.56)
Superficial wound revision			3	(0.23)
Haematoma evacuation			2	(0.15)
THA revision			6	(0.46)
	Isolated femoral head and acetabular liner exchange		1	(0.08)
	Isolated femoral component revision		4	(0.30)
		Short-stem to straight-stem implant	2	(0.15)
		Short-stem to femoral revision implant	2	(0.15)
	All component revision		1	(0.08)

SD—standard deviation; THA—total hip arthroplasty.

**Table 5:** Complications and revision procedures during follow-up.

		Study Sample (n = 1307)	
Follow-up			
Follow-up (months), median (IQR)		86.0	(48–130)
Follow-up (years), median (IQR)		7.0	(4–10)
Surgery-related complications during follow-up, n (%)		89	(6.81)
Periprosthetic joint infection (PJI)		3	(0.23)
	Acute PJI	1	(0.08)
	Chronic PJI	2	(0.15)
Mechanical complications		71	(5.43)
	Stem subsidence requiring revision	20	(1.53)
	Aseptic loosening femoral component	28	(2.14)
	Failure of neck adapter (modular stem)	3	(0.23)
	Aseptic loosening acetabular cup	10	(0.77)
	Bearing surface wear	3	(0.23)
	Instability, dislocation	2	(0.15)
	Periprosthetic fracture	5	(0.38)
Heterotopic ossification		5	(0.38)
Iliopsoas impingement		10	(0.77)
Revision procedures during follow-up, n (%)		71	(5.43)
Mean time to revision (months), median (IQR)		6	(2–14)
Isolated femoral head and acetabular liner exchange		10	(0.77)
	Aseptic head and liner exchange	9	(0.69)
	DAIR procedure	1	(0.08)
Isolated component revision		55	(4.21)
	Femoral component revision	51	(3.90)
	Acetabular component revision	4	(0.31)
All-component revision		4	(0.31)
	One-stage revision	1	(0.08)
	Two-stage revision	2	(0.15)
	Not specified	1	(0.08)
Osteosynthesis (ORIF, plate and cerclage wires)		2	(0.15)

DAIR—debridement, antibiotics, and implant retention; IQR—interquartile range; ORIF—open reduction and internal fixation; PJI—periprosthetic joint infection.

**Table 6:** Indications for stem revisions following primary short-stem THA.

Indications for Stem Revision	Inpatient Stay	Follow-Up	Total	
	n	n	n (%)	
Chronic PJI	–	2	2	(3.33)
Periprosthetic fracture	1	1	2	(3.33)
Stem subsidence	2	18	20	(33.33)
Aseptic loosening femoral component	–	28	28	(46.67)
Aseptic loosening acetabular cup	1	1	2	(3.33)
Dislocation	–	1	1	(1.67)
Failure of neck adapter	–	3	3	(5.00)
Leg length discrepancy	1	–	1	(1.67)
Not specified	–	1	1	(1.67)
Sum	5	55	60	(100.00)

PJI—periprosthetic joint infection.

## Data Availability

The data presented in this study are available on reasonable request from the corresponding author due to ethical and data privacy/protection reasons.

## Abbreviations

ASA	American Society of Anesthesiologists
BMI	Body Mass Index
DAIR	Debridement, antibiotics, and implant retention
FAI	Femoroacetabular Impingement
IPFF	Intraoperative periprosthetic femur fractures
LCPD	Legg–Calvé–Perthes disease
LLD	Leg length discrepancy
LTRR	Lifetime risk of revision
OA	Osteoarthritis
ORIF	Open reduction and internal fixation
PJI	Periprosthetic joint infection
PPFF	Postoperative periprosthetic femur fractures
rTHA	Revision total hip arthroplasty
THA	Total hip arthroplasty
SCFE	Slipped capital femoral epiphysis
